# “suMus,” a novel digital system for arm movement metrics and muscle energy expenditure

**DOI:** 10.3389/fphys.2023.1057592

**Published:** 2023-01-26

**Authors:** Teresa Gerhalter, Christina Müller, Elke Maron, Markus Thielen, Teresa Schätzl, Anja Mähler, Till Schütte, Michael Boschmann, René Herzer, Simone Spuler, Elisabetta Gazzerro

**Affiliations:** ^1^ Muscle Research Unit, Charité-Universitätsmedizin Berlin, Berlin, Germany; ^2^ Experimental and Clinical Research Center, a joint Cooperation between the Max Delbrück Center for Molecular Medicine in the Helmholtz Association and the Charité-Universitätsmedizin Berlin, Berlin, Germany; ^3^ Charité-Universitätsmedizin Berlin, Corporate Member of Freie Universität Berlin and Humboldt-Universität zu Berlin, Berlin, Germany; ^4^ Basebox.Health, Utting, Germany; ^5^ ElanPhysio, Berlin, Germany; ^6^ Max Delbrück Center for Molecular Medicine in the Helmholtz Association (MDC), Berlin, Germany; ^7^ Clinical Study Center (CSC), Berlin Institute of Health at Charité–Universitätsmedizin Berlin, Berlin, Germany

**Keywords:** accelerometers, energy expenditure, neuromuscular diseases, inertial sensors, muscular dystrophies, outcome measures, smartwatch, apple watch

## Abstract

**Objective:** In the field of non-treatable muscular dystrophies, promising new gene and cell therapies are being developed and are entering clinical trials. Objective assessment of therapeutic effects on motor function is mandatory for economical and ethical reasons. Main shortcomings of existing measurements are discontinuous data collection in artificial settings as well as a major focus on walking, neglecting the importance of hand and arm movements for patients’ independence. We aimed to create a digital tool to measure muscle function with an emphasis on upper limb motility.

**Methods**: suMus provides a custom-made App running on smartwatches. Movement data are sent to the backend of a suMus web-based platform, from which they can be extracted as CSV data. Fifty patients with neuromuscular diseases assessed the pool of suMus activities in a first orientation phase. suMus performance was hence validated in four upper extremity exercises based on the feedback of the orientation phase. We monitored the arm metrics in a cohort of healthy volunteers using the suMus application, while completing each exercise at low frequency in a metabolic chamber. Collected movement data encompassed average acceleration, rotation rate as well as activity counts. Spearman rank tests correlated movement data with energy expenditure from the metabolic chamber.

**Results:** Our novel application “suMus,” sum of muscle activity, collects muscle movement data plus Patient-Related-Outcome-Measures, sends real-time feedback to patients and caregivers and provides, while ensuring data protection, a long-term follow-up of disease course. The application was well received from the patients during the orientation phase. In our pilot study, energy expenditure did not differ between overnight fasted and non-fasted participants. Acceleration ranged from 1.7 ± 0.7 to 3.2 ± 0.5 m/sec^2^ with rotation rates between 0.9 ± 0.5 and 2.0 ± 3.4 rad/sec. Acceleration and rotation rate as well as derived activity counts correlated with energy expenditure values measured in the metabolic chamber for one exercise (r = 0.58, *p* < 0.03).

**Conclusion:** In the analysis of slow frequency movements of upper extremities, the integration of the suMus application with smartwatch sensors characterized motion parameters, thus supporting a use in clinical trial outcome measures. Alternative methodologies need to complement indirect calorimetry in validating accelerometer-derived energy expenditure data.

## Introduction

Real world evidence and patient-related outcome measures (PROMS) are increasingly replacing artificial functional tests for outcome quantification in clinical trials (Marquis-Gravel et al., 2109; Goldsack et al., 2020; Mantua et al., 2021). Sensitive digital measures are of particular interest for genetic neuromuscular diseases, severe progressive degenerative disorders. These chronic conditions affect an estimated number of 500.000 patients in Europe, leading to lifelong disability and imposing a significant burden on families and healthcare system (Bhatt, 2016; Ryder et al., 2017; Dangouloff et al., 2021).

Muscular dystrophies present with muscle weakness and atrophy, with progressive reduction in strength and limitation of motor function leading to wheelchair dependence, loss of arm motility and, often, need for respiratory support.

After having been regarded as untreatable disorders, leaving room for physiotherapy as unique care intervention, the field is now facing major therapeutic advances and first causative therapies. Stem cell technologies and the utilization of CRISPR/Cas9 tools for gene editing are leading to justified hope for treatment options in muscular wasting disorders. Exon skipping for Duchenne Muscular Dystrophy has been developed (Takeda et al., 2021; Wilton-Clark and Yokota, 2022) and mRNA-based precise and safe mutation correction is suitable in primary human muscle stem cells (Escobar et al., 2021; Stadelmann et al., 2022).

However, together with therapeutic developments, there is a great need to improve clinical trial designs in terms of speed, relevance, and costs. The current outcome measures standardized for assessing new treatments in genetic muscle diseases are mostly focused on lower limb motility and are tested in artificial hospital setting. Examples for widely used motor scales are timed function tests (i.e., time to stand from supine, time to run or walk 10 m, time to climb four stairs), the North Star Ambulatory Assessment (NSAA) score and quantitative muscle testing (Pane et al., 2012). The distance covered in 6 min (six-minute walk test) is also a well-known validated example (Mah et al., 2022). Complementary to functional scales, muscle magnetic resonance imaging is sensitive to depict subtle changes in fat replacement of muscles and can provide patterns of muscle involvement. However, it is costly and assesses muscle tissue quality at single time points (Burakiewicz et al., 2017; Naarding et al., 2020).

In less than half of currently recruiting or active trials for muscular dystrophies (http://www.clinicaltrials.gov, September 2022), primary endpoints include upper limb function. Non-ambulant patients are then more easily excluded from recruitment, although upper-limb-motility is crucial for quality of life and independence (Schuster et al., 2022). In the case that upper limb strength is part of the outcome measures, this consists of observer performance in a controlled medical environment and questionnaires (Mayhew et al., 2013; Domingos and Muntoni, 2018). These tests are not able to quantify changes in small movements, neither to extrapolate daily fluctuations in motor function nor to provide information on patient-related tasks or PROMS.

Triaxial accelerometers loaded on activity trackers or smartwatches are effective and promising tools in monitoring movement disorders in both clinical and research settings (Varghese et al., 2021). Movement data derived from accelerometers can also extrapolate energy expenditure (EE) and values of physical activity. Once again, most of the studies focused on lower limbs (e.g., treadmill, running trials, cycling) (Freedson et al., 2005; Crouter et al., 2006; Dooley et al., 2017; Zhang et al., 2019; Jeng et al., 2020). It is not feasible to apply these data for assessing upper limb function, since regression equations based on lower limb parameters underestimate EE quantification in non-locomotive activities (Bassett et al., 2000; Murakami et al., 2019; Fernández-Verdejo et al., 2021).

Thus, there is still an unmet need for a standardized digital system able to provide real-world upper limb movement metrics and quantification of physical muscle activity for patients affected by neuromuscular diseases. We therefore created the digital application (App) “suMus (sum of muscle activity).” suMus utilizes inertial sensors loaded on an Apple Watch, thus providing continuous reading of arm movements. In a first orientation phase, 80 standardized exercises were tested by 50 patients affected by different genetic muscular disorders over a seven-month period.

In a second step, we validated suMus derived motility parameters in healthy subjects in a metabolic chamber, to correlate accelerometer-derived measures of upper limb movements with EE.

## Materials and methods

### Study design

The study was structured in three sequential stages:

1. Designing and programming of the suMus research smartwatch App and the suMus web-based platform.
2. Orientation phase, 50 patients affected by different genetic neuromuscular disorders trained at home and tested different exercises from a pool of 80 activities *via* the suMus web-based platform.
3. Validation phase of the suMus App, i.e., movement analysis in healthy subjects.

### Study cohort

For the orientation phase, patients were recruited from the Outpatient Clinic for Muscular Diseases at the Experimental and Clinic Research Center (ECRC) at Charité, Berlin and by information through patients’ organization groups (http://www.dgm.org [LGMD-group]). Before inclusion, all patients or their parents provided signed informed consent. The study included ambulant and not ambulant patients older than age of 14 years and with genetic confirmation of a neuromuscular disease. No specific exclusion criteria were introduced.

For the movement analysis healthy volunteers were recruited at our institution.

### Study protocol

A randomized protocol of four exercises was completed in a metabolic chamber while wearing the suMus-Apple watch on the left wrist.

The metabolic chamber is a comfortable, airtight room (width: 2.5 m, depth: 2.0 m, height: 2.2 m) that is constantly supplied with fresh air like an open circuit indirect calorimeter. Carbon dioxide (CO_2_) production and oxygen (O_2_) consumption were measured to calculate resting and exercise EE, as previously described (Mähler et al., 2012).

#### Activity protocol

Subjects were tested in groups of three per day (early morning, late morning, early afternoon). The first subject of the day was analyzed after a 12 h overnight fast, the second and the third subjects had no dietary restrictions.

Clinical history and anthropometric measurements (body mass index, blood pressure, and heart rate) were recorded before starting the exercises. A questionnaire focused on caloric intake and physical activity of the day preceding the examination was fulfilled.

Whilst being seated in a comfortable chair in the metabolic chamber measurements were started at first for air equilibration (30 min) and then for assessing subjects resting EE (30 min). Then, subjects started the four exercises according to a protocol in a randomized order including alternating arm elevation at 180° (exercise 1: “cherry picking”), parallel arm elevation at maximal 45° (exercise 2: “little flyer”), alternate anterior arm extension at shoulder height (exercise 3: “boxing”), and hand biking on an ergometer (exercise 4) (Theravital Medica GmbH, DE).

In exercises 1-3, a metronome set at 15 beats per minute regulated the frequency of repetitions, while the speed of the movement was left free. The hand-ergometer was adjusted to a low workload of 0.02 W/kg body weight with a speed of 40–45 rounds per min.

Each exercise had to be performed for 10 continuous minutes due to the sampling frequency of the metabolic chamber. Between exercises, subjects completely rested for 10 min to prevent interference among the different movements.

**FIGURE 1:**
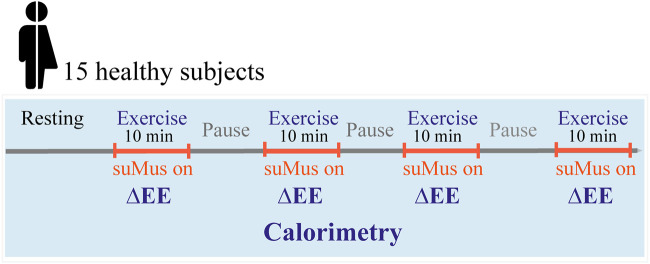
Study design for calorimetry and movement measuements. Subjects performed four different exercises in the metabolic chamber with defined resting periods. Movement measurement were recorded during exercises indicated in orange, while the metabolic chamber recorded also during the resting periods. ΔEE: difference of energy expenditure between exercise and pause phase.

The suMus App was activated by the subjects only at the beginning of each single exercise. Through video-monitoring researchers tracked the time of each exercise and provided verbal cues to the participants to transition to next phase. The difference between absolute values of exercise EE and resting EE (ΔEE) was calculated every 10 min between each exercise (Figure 1).

### suMus smartwatch app

suMus provides a custom-made App written in Objective-C that runs on the Apple smartwatch as well as on an iPhone connected to the watch *via* Bluetooth, which is available on Apple’s AppStore. Movement data and feedbacks are collected by suMus with a Universally Unique Identifier (UUID). Per default, data are transferred to the iPhone and are available in Apple’s Health and Activity App-HealthKit (https://developer.apple.com/documentation/healthkit/data_types) but are not sent to an Apple server. When connected to WLAN, common network standards (HTTPS, JSON, REST) send the data attitude quaternion (x, y, z, w), rotation rate (x, y, z), gravity (x, y, z), user acceleration (x, y, z), magnetic field (x, y, z, c), heading (angle) directly from the HealthKit-App to a suMus server. Data are hence stored in the subject’s specific folder in the backend of the suMus web-based platform, from which they can be extracted as CSV data (Supplementary Figure S1).

The suMus server-side application is implemented in Python using the Django web framework. It manages all patients and therapists and allows care givers to maintain patient-specific exercise plans on a weekly basis.

### Data processing

For quantification of EE, measured maximal Oxygen consumption VO2 data (mL/min) were multiplied by 1,000 to obtain VO_2_ in L/min, and then multiplied by 4.867 kcals/L to obtain kcals/min.

Movement data from the suMus-Apple Watch were continuously recorded with a 20 Hz sampling frequency rate. We set the starting point of analysis of the raw data at the beginning of a rhythmic movement. The shortest execution was for 8 min and twenty second; for uniformity of data points, we cut all the exercises for this duration. CSV files were processed in Matlab (MathWorks, Natick, MA, United States) to extract acceleration in meter per square second (m/sec^2^) and the rotation rate in radians per sec (rad/sec).

To complete a linear regression analysis of accelerometer-predicted EE values *versus* measured-EE, acceleration data were converted to activity counts per 1 s epochs.

For exercises 1, 2, 3, given the high interference noise signal, activity counts were calculated for each participant by inserting a variable threshold at mean acceleration plus two times the standard deviation (SD). For exercise 4, in which no noise signal was detected because of continuity of the movement on the hand-ergometer, the threshold was fixed at 0.05 m/sec^2^ for each subject.

For all exercises, low activity counts were defined as values above the threshold, medium activity counts were defined as values higher than two times the threshold.

### Statistical analysis—Movement analyses

Results were expressed as mean ± SD. Statistical analysis was performed in Matlab (MathWorks, Natick, MA, United States). To evaluate the relative dispersion of data points in a data series around the mean, the coefficient of variation was calculated (CV). Differences among exercises were tested with *t*-test. Correlation between parameters was analyzed with Spearman’s rank correlation coefficient.

A *p*-value <0.05 was considered significant for all statistical tests.

### Data availability

Anonymized data not published within this article will be made available by request from any qualified investigator.

### Ethic approval

The study was approved by the Local Ethics Committee of Charité Universitätsmedizin Berlin (EA1/212/21).

## Results

### suMus smartwatch app

**FIGURE 2:**
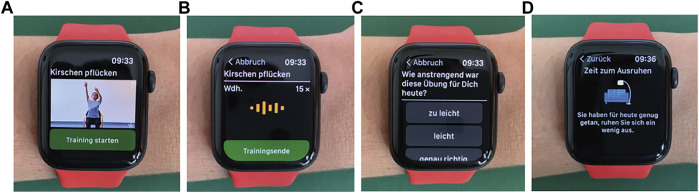
Programming of the suMus App: **(A)** Tailored exercise plans designed by the therapists are visualized in the App; **(B)** During the training, sensors register acceleration and rotation rate; **(C)** After the exercise, the patient is asked to provide feedbacks; **(D)** After completing the whole exercise plan, patients are informed through a visual input that rest is suggested.

The suMus App contains 80 exercise videos for upper/lower extremities, trunk, and respiratory muscles. On the individual suMus profiles, patients can visualize their customized exercise plan designed by their own therapists for the incoming weeks, the corresponding instructional video, and the exercise details (number of repetitions per day and per week and) (Figure 2A). During executing the task, the watch inertial sensors register movement (Figure 2B).

After completing the exercise, patients are asked to answer questions on difficulty level, whether they had pain during/after the exercises and whether they had fun (Figure 2C). Once having completed the whole daily training plan, patients are informed through a visual input that rest is suggested since excessive movement could be damaging (Figure 2D).

The movement data and feedbacks are sent to patient’s specific folder in the suMus web-based platform. Here, through a therapist profile, therapists/caregivers can visualize them and adjust accordingly the exercise plans for the following weeks (Supplementary Figure S1).

#### suMus web-based platform (https://sumus.digital) and orientation-phase

The acceptance profile of the suMus exercises and the accessibility of the suMus web-based platform were initially tested by 50 patients affected by different genetic muscle disorders, who agreed to perform daily physical training according to their personalized suMus exercise plans at home for seven-months. Through the platform, the patients were able to confirm to their physiotherapists the execution of the weekly training plan and to provide feedbacks. Acceptance was monitored through weekly feedbacks and one questionnaire (Supplementary Figure S3).

Muscular dystrophies were the most represented disease types in the patient population register. They included 23 patients with limb girdle muscular dystrophies and 13 with facioscapulohumeral muscular dystrophy. Additionally, the study included congenital and myofibrillar myopathies, myotonic dystrophy type I, motoneuronal disorders, one McArdle’s disease and one hereditary sensorimotor neuropathy (Supplementary Figure S2A).

**FIGURE 3:**
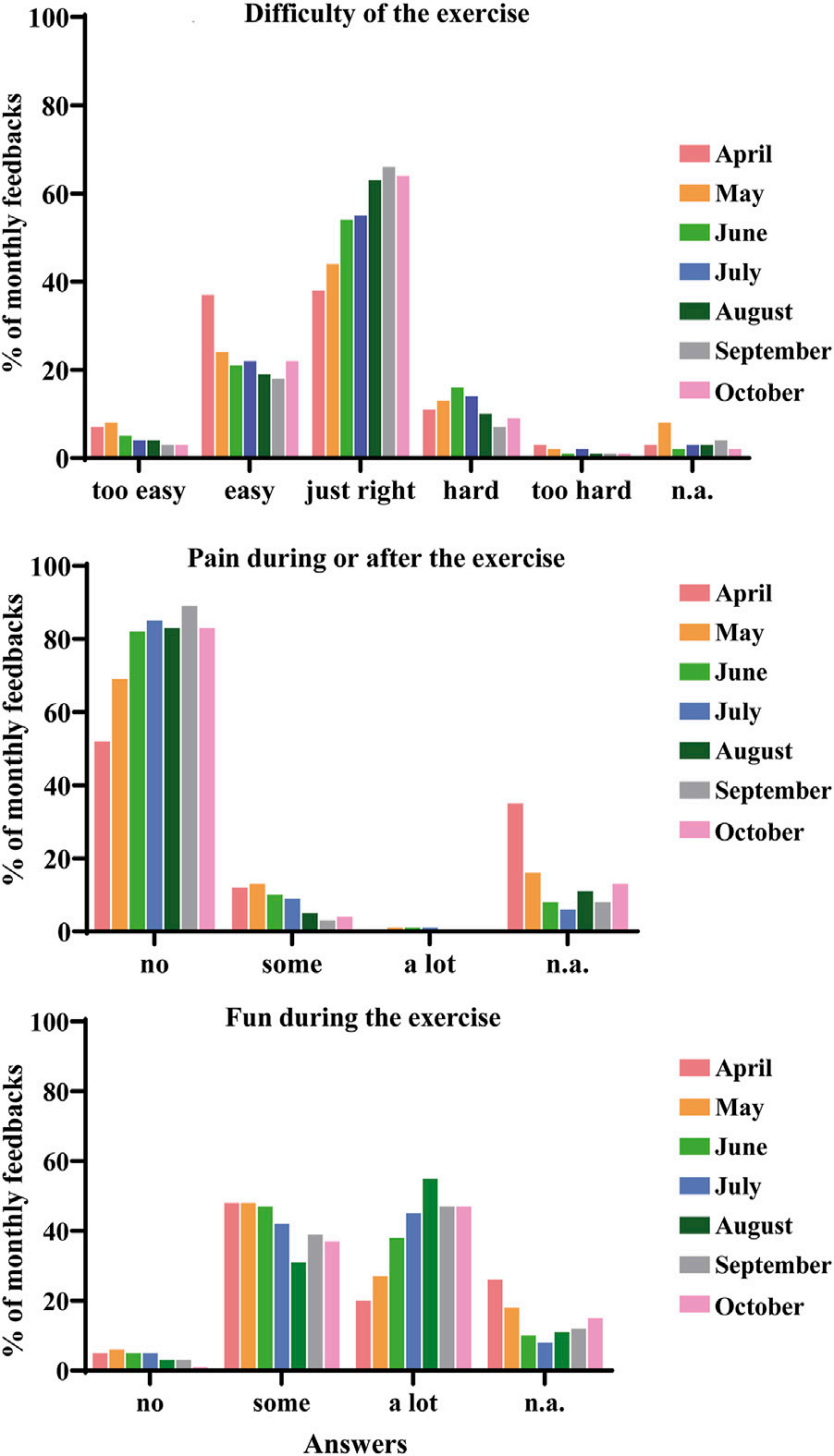
Monthly feedbacks on difficulty, pain, and fun levels by the cohort of patients in the seven-month period of orientation phase.

The monthly monitoring of the feedbacks showed that the adherence of the suMus exercises to the patients’ individual needs and the efficacy of the patients’ physiotherapists communication gradually improved over time, as the feedback “just right” and “I had no pain” raised from 38% to 64% and 52% to 83%, respectively, along the study. Similarly, the fun factor increased from 20% to 47% (Figure 3).

During this orientation phase, we recorded for each exercise the frequency of execution, the number of feedbacks “the exercise was just right for me” and “I had no pain here.” The movements “cherry picking” (exercise 1), “little flyer “(exercise 2), and “boxing” (exercise 3) displayed a very high acceptance profile and were hence selected for the validation of the suMus smartwatch App in the upper extremities (Supplementary Figure S2B, Supplementary Videos S1–S3). As a gold standard for quantifying exercise, we added hand biking on a hand-ergometer (exercise 4).

### Movement analysis—Validation of the suMus smartwatch app

Fifteen healthy participants (6 men and 8 women), aged 20–55 years, completed the randomized protocol of four exercises in a metabolic chamber while wearing the suMus Apple watch on the left wrist. None of the participants had chronic diseases that could affect their metabolism or daily physical activity. Participant anthropometric characteristics are summarized in Table 1.

**TABLE 1:** Characteristics of the 15 participants of the movement study.

Characteristics	Exercise cohort (n =15)
Age (years)	34.6 ± 9.2
Gender	7F/8M
Height (cm)	171 ± 10.5
Weight (kg)	72 ± 12
BMI (kg/m^2^)	24.8 ± 4.2

Data are given as mean ± SD; BMI, body mass index.

All subjects except one were able to complete every task. Accelerometer-derived measurements were correctly exported, saved in the suMus web-based platform, and extracted for analyses (Supplementary Figure S4).

**FIGURE 4:**
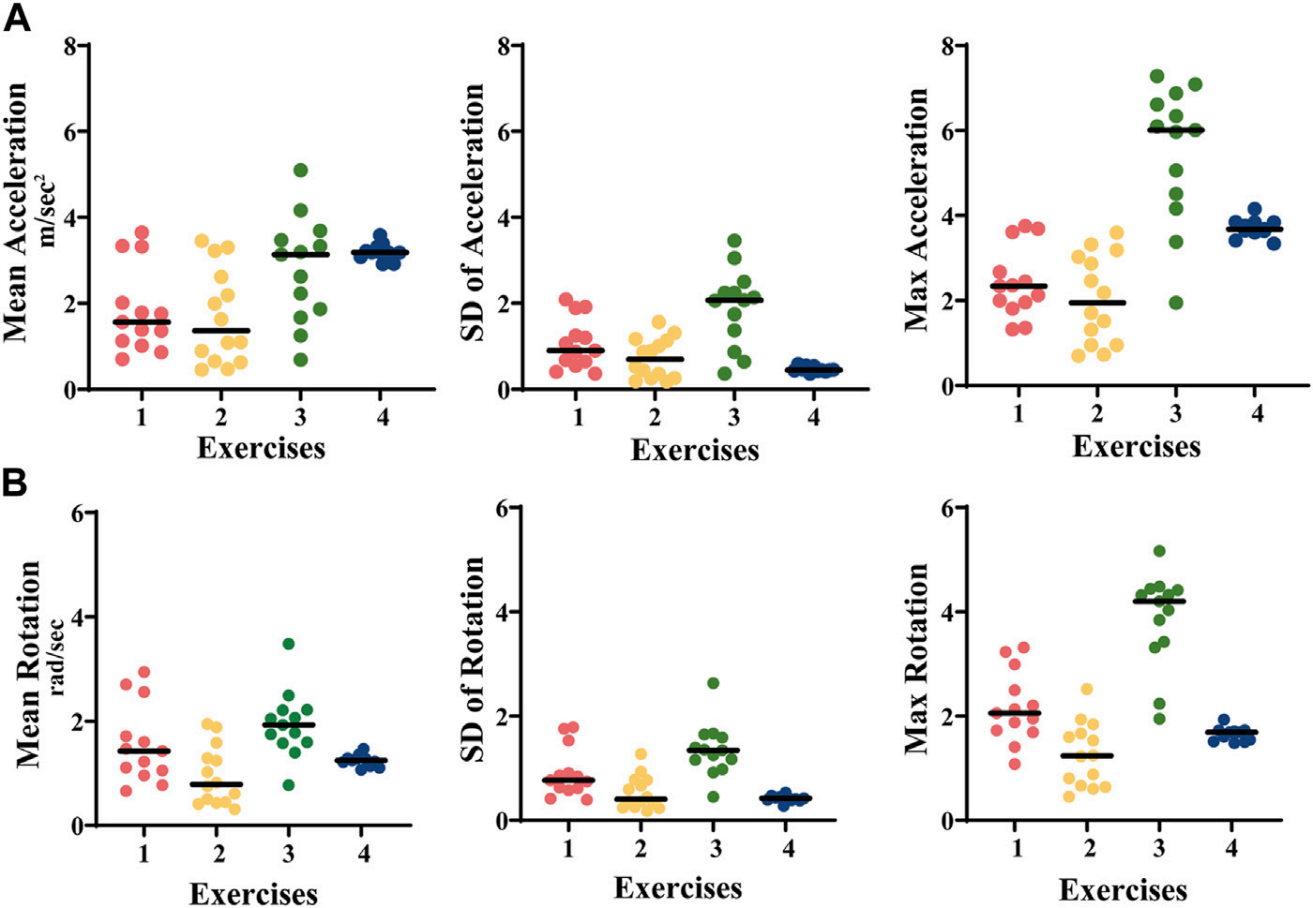
**(A)** Acceleration (m/sec^2^) and **(B)** rotation rate (grad/sec) during the four exercises in 14 healthy controls. Exercise 1: cherry picking, exercise 2: little flyer, exercise 3: shadow boxing, exercise 4: hand biking.

Figure 4 shows the distribution of acceleration and rotation rate in the 14 subjects according to the four exercises.

Exercises 1-3 displayed a higher distribution of the average, SD, and maximum values in comparison to exercise 4, in which the movement was guided by the instrument and with a fixed speed (Acceleration, mean ± SD: ex.1: 1.8 ± 1.1 m/sec^2^; ex.2: 1.7 ± 0.7 m/sec^2^; ex.3: 2.0 ± 1.9 m/sec^2^; ex.4: 3.2 ± 0.5 m/sec^2^. Rotation rate, mean ± SD: ex.1: 1.6 ± 0.9 rad/sec; ex.2: 0.9 ± 0.5 rad/sec; ex.3: 2.0 ± 3.4 rad/sec; ex.4: 1.2 ± 0.4 rad/sec) (Table 2).

**TABLE 2:** Acceleration (m/sec^2^) and rotation (Grad/sec) values during the four exercises in the 14 healthy subjects.

Acceleration	Exercise 1	Exercise 2	Exercise 3	Exercise 4
Mean	1.8	1.7	2.0	3.2
SD	1.1	0.7	1.9	0.5
CV	0.57	0.43	0.66	0.15
Max value	2.4	2.0	5.5	3.7

Rotation	Exercise 1	Exercise 2	Exercise 3	Exercise 4
Mean	1.6	0.9	2.0	1.2
SD	0.9	0.5	1.4	0.4
CV	0.58	0.56	0.67	0.33
Max value	2.2	1.3	3.9	1.7

Data are expressed as average of the means, SDs, CVs, and maximal (Max) values.

**FIGURE 5:**
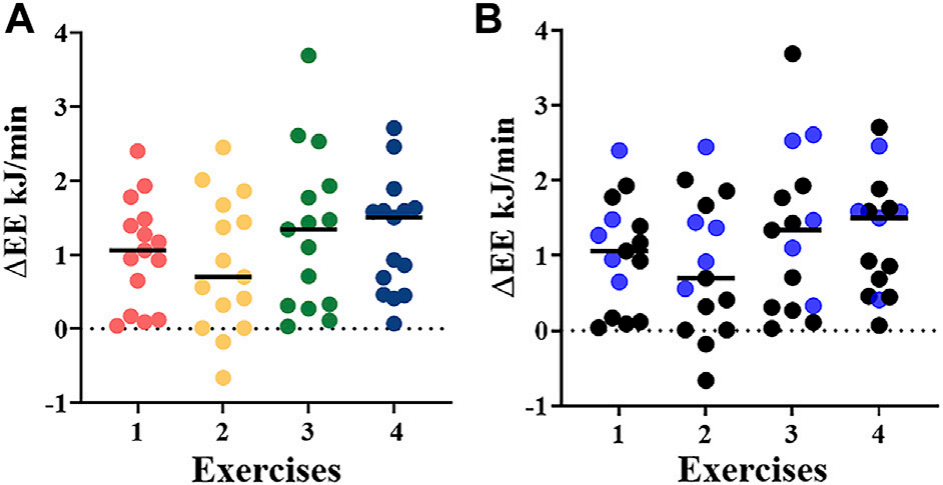
**(A)** ΔEE (KJ/min) derived from indirect calorimetry using the metabolic chamber during the four different exercises in 14 healthy controls. Exercise 1: cherry picking, exercise 2: little flyer, exercise 3: boxing, exercise 4: hand biking; **(B)** Differentiation of ΔEE (kJ/min) during the four different exercises in 5 (blue dots) and 10 (black dots) healthy controls with and without overnight fasting, respectively.

Figure 5A illustrates the ΔEE distribution in the 14 subjects according to the four exercises as assessed by indirect calorimetry. The low ΔEE values are in accordance with the low frequency of repetitions during the four exercises (mean ± SD: ex.1: 1.03 ± 0.72; ex.2: 0.86 ± 0.91; ex.3: 1.31 ± 1.07; ex.4: 1.25 ± 0.78). The CVs of the ΔEE data for exercise 1 is 0.7, for exercise 2 is 1.06, for exercise 3 is 0.83, and for exercise 4 is 0.64 (Table 3).

**TABLE 3:** ΔEE (KgJ/min) during the four exercises in the 14 healthy controls.

	Exercise 1	Exercise 2	Exercise 3	Exercise 4
Mean	1.03	0.86	1.31	1.25
SD	0.72	0.91	1.07	0.78
Max value	2.40	2.45	3.69	2.71
Min value	0.04	−0.06	0.03	0.07
CV	0.70	1.06	0.82	0.62

Data are expressed as means, SDs, maximal (Max) and minimal (Min) values.

No significant differences in ΔEE was identified among the individual four exercises and between overnight fasted (*n* = 5) and non-fasted (*n* = 10) participants (Figure 5B).

**FIGURE 6:**
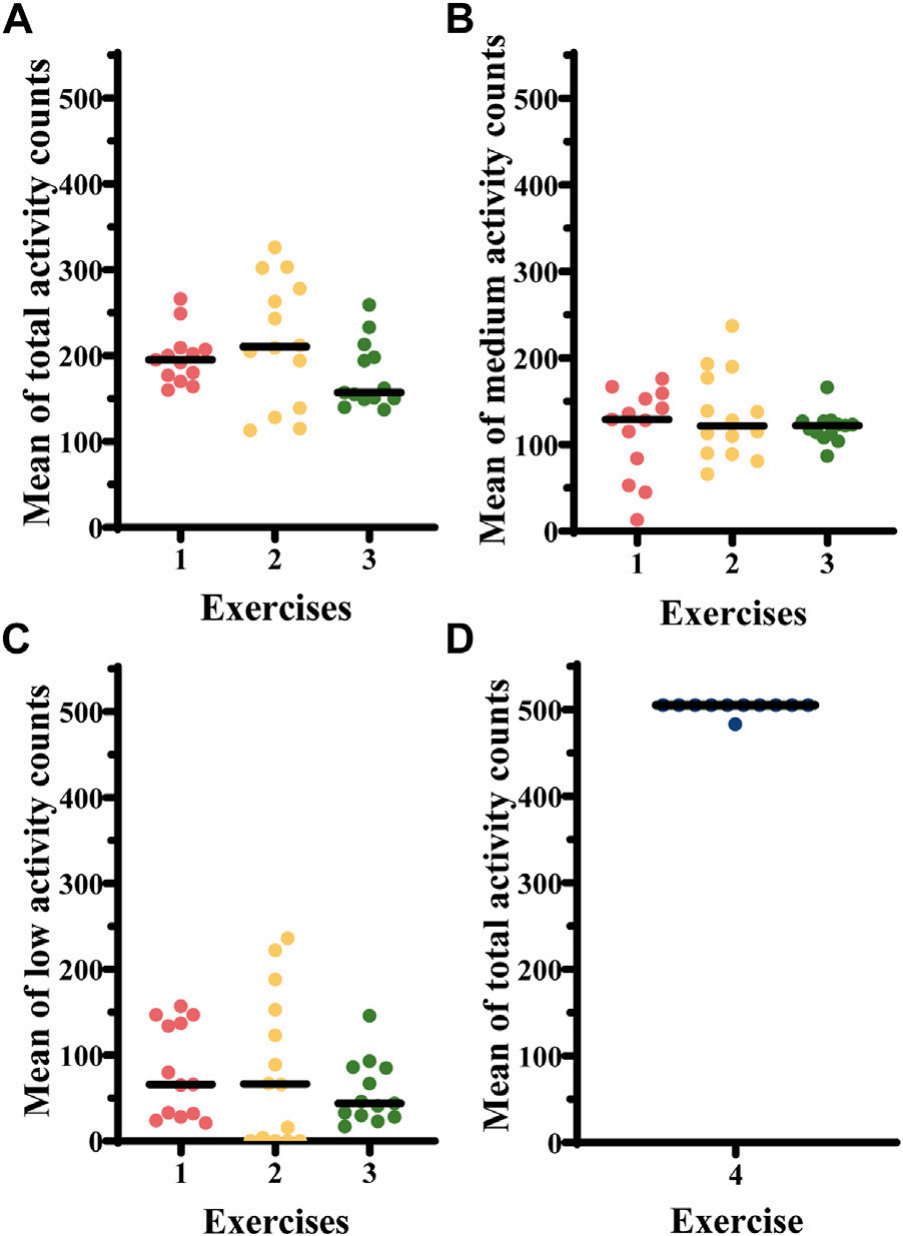
**(A)** Total activity counts, **(B)** medium activity counts, and **(C)** low activity counts derived from the accelerometry data for free arm movement exercises 1–3 in 14 healthy controls. **(D)** total activity counts for exercise 4 in 14 healthy controls. Exercise 1—cherry picking, exercise 2—little flyer, exercise 3—shadow boxing, exercise 4—hand biking.

For exercises 1–3, we converted acceleration data to total, medium and low activity counts (Figures 6A–D). Among the 14 participants, the low counts (the data points just above the variable threshold, mean acceleration + two times SD) displayed in all four groups a CV between 0.65 and 1.05. In exercise 4, the values of total activity (data points above the fixed threshold of 0.05 m/sec^2^) were highly homogenous showing a CV of 0.01 (Table 4).

**TABLE 4:** Total, medium, and low activity counts during each individual exercise in the 14 healthy subjects.

Exercise 1	Total	Medium	Low
Mean	197.8	115.4	82.4
SD	31.1	51.2	54.2
CV	0.17	0.44	0.66
Max value	266	176	157

Exercise 2	Total	Medium	Low
Mean	216.4	133.3	83.1
SD	72.9	49.5	87.3
CV	0.34	0.37	1.05
Max value	326	237	236

Exercise 3	Total	Medium	Low
Mean	176.8	119.9	56.9
SD	38.9	18.0	37.0
CV	0.22	0. 5	0.65
Max value	259	156	146

Exercise 4	Total		
Mean	503.1		
SD	6.3		
CV	0.01		
Max value	505		

Data are expressed as means, SDs, CVs, and maximal (Max) values.

**FIGURE 7:**
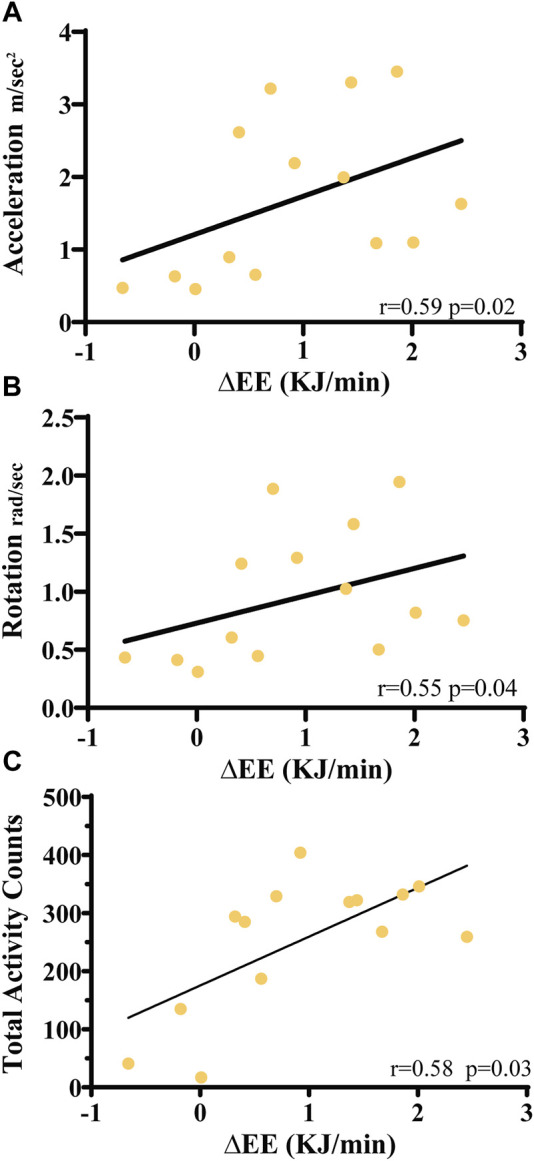
Linear regression analysis of ΔEE values with **(A)** average acceleration values, **(B)** average rotation levels and **(C)** acceleration-derived average total activity counts for exercise 2. Coefficients of determination (r2) and the statistical significance *p*-value are indicated.

The relationship between ΔEE measured in the metabolic chamber, accelerometer-derived activity counts, acceleration and rotation metrics was then evaluated through linear regression analysis. Exercise 2 (Figure 7) yielded a statistically significant correlation (r = 0.58, *p* < 0.03). A higher score of linear regression was observed at higher ΔEE values. For exercise 1, 3, and 4 no linear correlation was identified (data not shown).

## Discussion

We present suMus, a new digital endpoint for upper limb movement metrics in genetic muscle disorders. SuMus consists not only of an innovative App using inertial sensors of a smartwatch but also of a closed-loop feedback ecosystem involving patients, physiotherapists, and physicians, plus highest-standard data protection measures.

We first tested suMus strategy and feasibility in 50 patients affected by distinct muscle genetic disorders during a seven-month orientation phase. The patients’ feedbacks confirmed easy accessibility of the tool, good adherence of the proposed exercises to patients’ motoric profiles and improved communication with the therapists.

We next validated suMus movement sensors for the characterization of upper limb movements in healthy volunteers. They completed a protocol of four exercises at a precise slow frequency in a metabolic chamber for parallel assessment of indirect calorimetry. Indeed, the suMus algorithm aims to extrapolate EE data from movement metrics to display them to patients as well as to apply them as endpoints in observational/interventional clinical trials. In neuromuscular disorders, the primary genetic defect is aggravated by secondary metabolic alterations, which affect muscle cells energy production and flexibility, leading to movement-induced muscle fatigue (Lindsay et al., 2019; Peric et al., 2019). Patients with muscular dystrophies display a less efficient pattern of systemic energy exchange than healthy individuals (Boschmann et al., 2010 and Boschmann et al. unpublished data). A precise quantification of the impact of new therapeutic approaches on such energy abnormalities would constitute a relevant additional outcome in clinical studies.

The use of suMus proved to be easy and intuitive for all but one participant.

The accelerometer-derived signals resulted in quantifiable arm metrics and were able to differentiate in exercises 1-3 a comprehensive range of acceleration and rotation measures, while they provided a uniform set of data in exercise 4, where the speed of movement was fixed.

We measured the ΔEE between the four 10-min exercises and 10 min-pause phases in volunteers without any previous dietary restriction. Consistently with our protocol including only slow frequency arm movements without any changes in body posture and activity of the lower limbs, the resulting ΔEE values were low with means ranging from 0.86 to 1.31 kJ/min. In previous studies we had quantified absolute EE in overnight fasted subjects performing a single 40-min long exercise after a resting phase of 30 min (Mähler et al., 2012). Hence, to control for the impact of postprandial processes on ΔEE, five additional volunteers fasted in the 12 h preceding the study. Interestingly, the quantification of the exercise-pause ΔEE blunted the impact of post-prandial thermogenesis, as we did not detect a statistical significance difference between the fasted and not fasted groups.

Multiple reports developed regression equations to correlate accelerometer-extrapolated EE and indirect calorimetry ΔEE values but have provided inconsistent results (Freedson et al., 2005; Crouter et al., 2006; Dooley et al., 2017; Roskoden et al., 2017). Previous analyses, focused on upper extremities, measured arm motion and EE in the context of various simulated free-living occupations, including a wide range of indoor and outdoor movements of variable intensity, with transition in body positions and directions, leading to possible controversial conclusions (Roskoden et al., 2017; Murakami et al., 2019; Kwon et al., 2021).

In the present study, the mean of 10 min accelerometer-derived motion data (acceleration, rotation, activity counts) and the corresponding ΔEE values were tested for correlation coefficient, which resulted significant only in exercise 2 (parallel arm elevation at maximal 45°). We link the limited correlation to the small range and small number of data points. In our protocol, we excluded high and medium frequency movements. Rather, we focused on validating the threshold of signal acquisition by the accelerometers in slow frequency activity as this is the only one feasible for patients affected by neuromuscular diseases. Secondly, the inclusion in the analysis of three subgroups of subjects in a very different metabolic state (overnight fasted, not fasted in the morning, after lunch in the early afternoon) caused a high dispersion in the measured ΔEE data and therefore a weaker correlation with accelerometer-predicted values. However, our goal was the assessment of activity tracking under normal conditions of daily life. Finally, we correlated two methods characterized by a very different temporal accuracy. The accelerometer inertial sensors transferred a detectable signal every 0.05 s, while the metabolic chamber is limited to a 10-min temporal resolution and provided one single EE value for each exercise (Chen et al., 2018). Thus, we could not analyze data from shorter interval periods (2 min, for example) and then apply additional statistical models such as a multilevel correlation analysis (Grittner and Lahmann, 2015).

An alternative approach for our indirect calorimetry quantification could constitute the canopy breath-by-breath method using facemasks. This system has a minimum of 15 s of data processing and is therefore suitable to the analysis of rapid changes in the dynamic of metabolic signals such as in subjects performing exercises (Schoeffelen and Plasqui, 2018). We nevertheless chose a metabolic chamber for the first proof of suMus in healthy volunteers as we aimed to validate a method, which could have been realistically adopted in following studies in patients with neuromuscular diseases. The atrophy of the facial musculature and the restrictive ventilatory insufficiency, which can often affect these patients, render the use of a tight facemask rather impracticable, while the near free-living environment of a metabolic chamber enables various types of studies.

Alternative approaches for arm movement tracking in neuromuscular diseases were previously introduced. First, the stereo camera-based reachable workspace analysis, currently adopted as endpoint in clinical trials for facioscapulohumeral muscular dystrophy, measures relatively simple well visible movements to be performed inside the precise working volume of the device. The system is nevertheless restrained by a low efficiency in capturing free motion and is limited by significative ceiling and floor effects, which exclude the recruitment of patients with respectively light or severe form of disease. A similar bias is displayed by Microsoft-Kinect gaming interfaces associated to skeleton tracking algorithms (Han et al., 2016; Heutinck et al., 2018; Alfano et al., 2020; Gotthelf et al., 2021). Wearable sensors provided sensitive indicators of clinical changes in Duchenne Muscular Dystrophy and Spinal Muscle Atrophy (Le Moing et al., 2016; Chabanon et al., 2018; Annoussamy et al., 2021; Bouman et al., 2021). The stride velocity 95th centile, measured at the ankle by using the wearable-device Actimyo (Sysnav, France), received qualification from the European Medicine Agency as an acceptable secondary endpoint in clinical trials of ambulant patients affected by Duchenne Muscular Dystrophy (Servais et al., 2021). Upper limb activity (wrist acceleration, wrist rotation angle, elevation rate), measured by Actimyo in a two-year longitudinal study in type 2 and type 3 Spinal Muscle Atrophy patients, significantly decreased at 6 months of analysis, while the standardized muscle function scale (MFM32) detected a change at only 24 months (Annoussamy et al., 2021). In these approaches, however, the data are transferred to researchers only, without any patient involvement.

suMus differs from these digital tools, since the “patient perspective and empowerment” was the very starting point of our concept. Apps loaded on smartwatches can actively engage patients in studies by collecting their PROMS and sending them feedbacks on their daily motor function. The increasing popularity and affordability of these technologies offers a much higher usability profile in patients’ social environment than research-based activity monitors, known to suffer from low long-term patients’ compliance (Servais et al., 2021). This opens the possibility of extended acquisition of precise longitudinal data. Further, the smartwatch-technology is a proved relevant source of objective motility monitoring, allowing great precision in recording subtle changes in any patients’ home environment. In large cohorts of patients affected by Parkinson’s disease, smartwatch accelerometer data could quantify low hand-tremor amplitudes and frequencies with high accuracy (Hadley et al., 2021; Powers et al., 2021).

The strength of our study consisted in the application of a precise randomized exercise protocol exclusively designed for upper limbs and with controlled frequency of motion. However, our results confirm the limitations of prediction equations when applying accelerometer signals to EE estimation, particularly in the case of very light activities (Lyden et al., 2011; LaMunion et al., 2020). Our next strategy will be the collection of large amounts of personalized suMus accelerometer data to be analyzed by machine learning systems, able to identify and pattern patient’s physical activity profiles (Mardini et al., 2021).

**FIGURE 8:**
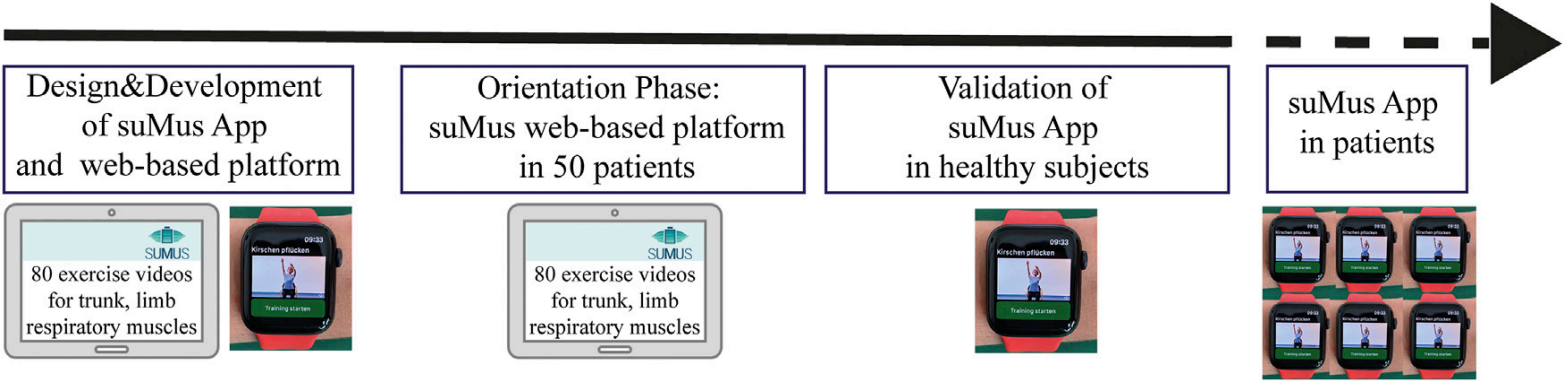
suMus Roadmap. Critical steps of suMus from concept to validation in patients.

Set of tailored exercises will be chosen in accordance with the specific patients’ motor function. Structured (exercise-based) and unstructured (free-style) movement data will be collected. The goals are to define individualized trajectory plots of disease course. The long-term acceptance of the suMus App at home, the reliability of the data over intervals of time, the sensitivity to detect clinical meaningful changes in correlation with standard outcome measures, the concordance of suMus metrics with patients PROMS and the development of learning algorithms for EE are our next research questions (Figure 8).

## Conclusion

suMus is a user-friendly, engaging, and digital sensor-based research system capable of providing arm metrics data in small frequency movements. Application in clinical trials addressing muscular dystrophies and other muscle wasting diseases is foreseeable.

This first exploratory study sets a baseline in healthy individuals. We will go further now to validate the suMus App in several patients affected by distinct genetic muscular disorders and different degree of muscle weakness.

## Data Availability

The raw data supporting the conclusion of this article will be made available by the authors, without undue reservation.

## Supplementary material

SUPPLEMENTARY FIGURE S1
**(A)** On the platform, the therapist creates weekly exercise plans for the patients and, **(B, C)**, can monitor their compliance to adjust the plan accordingly to the patients’ feedbacks.

SUPPLEMENTARY FIGURE S2
**(A)** Disease distribution of the patients in the orientation phase (*n* = 50) and **(B)** selection of the three exercises for the validation study based on the high acceptance. The feedbacks to the exercises obtained by patients using the suMus web-based platform in the period April-October 2021 were recorded and the three exercises for the upper limbs displaying a high acceptance profile (high number of executions, high number of answers “The exercise is just right” and “I had no pain during or after the exercise”) were selected. Abbreviations- FSHD, Facio-scapular-muscular-dystrophy; LGMD, Limb Girdle Muscular Dystrophy.

SUPPLEMENTARY FIGURE S3 Patients in the orientation phase provided feedback of their suMus experiences (*n* = 27). Their responses were collected via an optional, anonymised online-survey after they used the platform three months.

SUPPLEMENTARY FIGURE S4 Movement data obtained during the four exercises. Plotted are the device’s acceleration measured in increments of the gravitational acceleration, with the value 1 representing an acceleration of 9.8 m per second in the given direction of the watch. In addition, we recorded the rotation rate in radians per second around the device’s three primary axes.
