# Supplementary figures and images for: “suMus,” a novel digital system for arm movement metrics and muscle energy expenditure
Source: Front Physiol. 2023 Jan 26;14:1057592. doi: 10.3389/fphys.2023.1057592 (PMC9909604; doi:10.3389/fphys.2023.1057592)

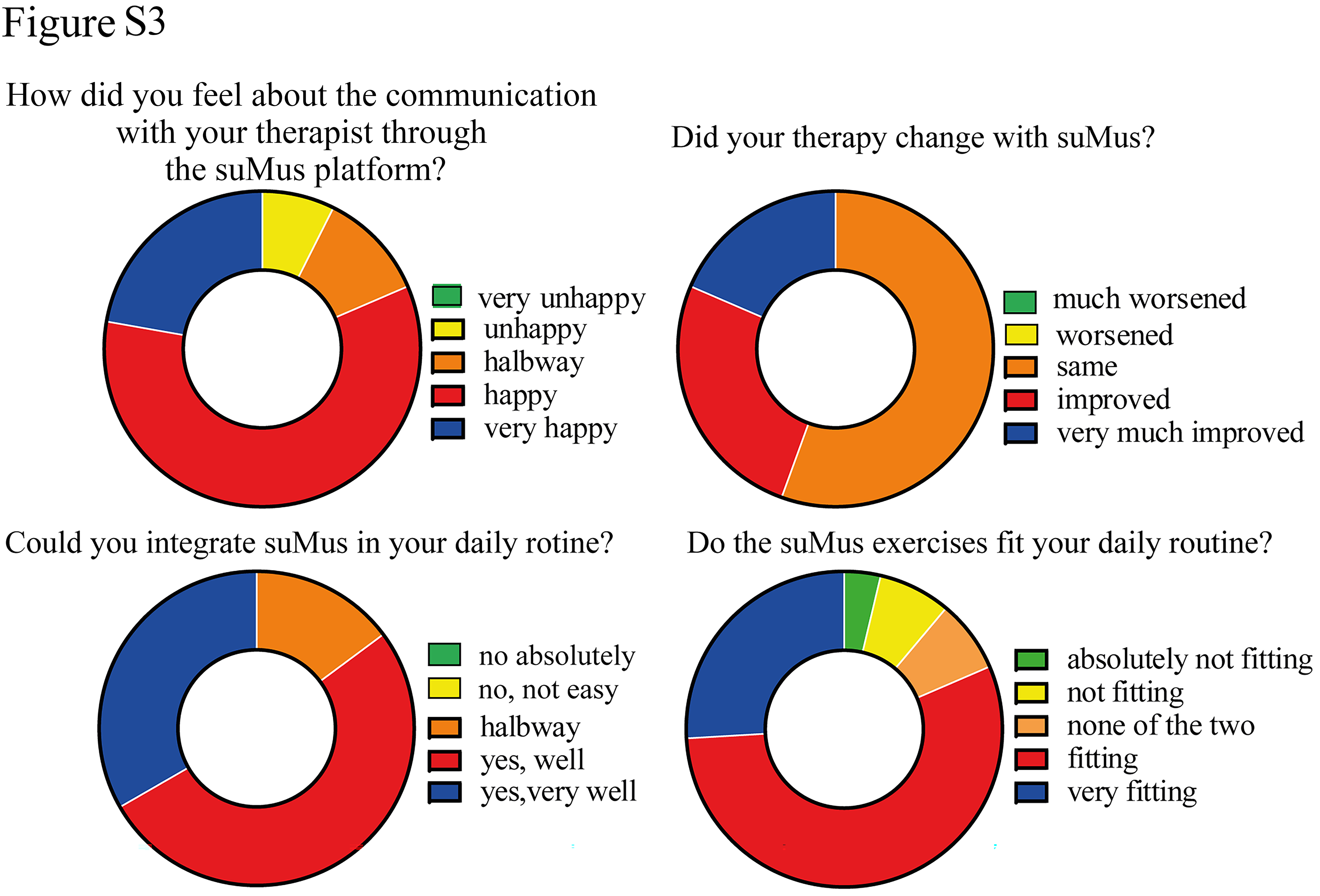

Supplement: Supplementary file 2 [file Image3.tif]

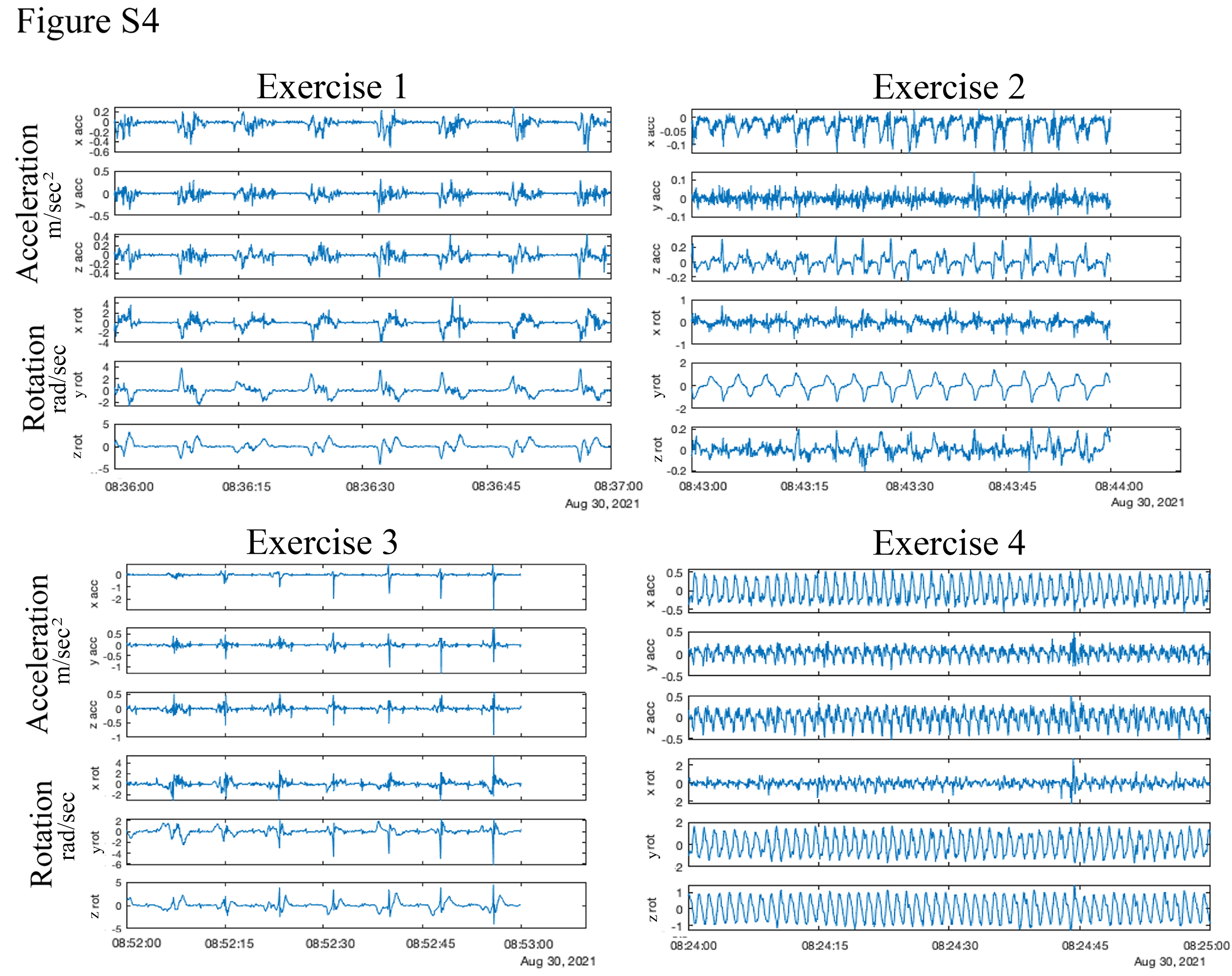

Supplement: Supplementary file 3 [file Image4.TIF]

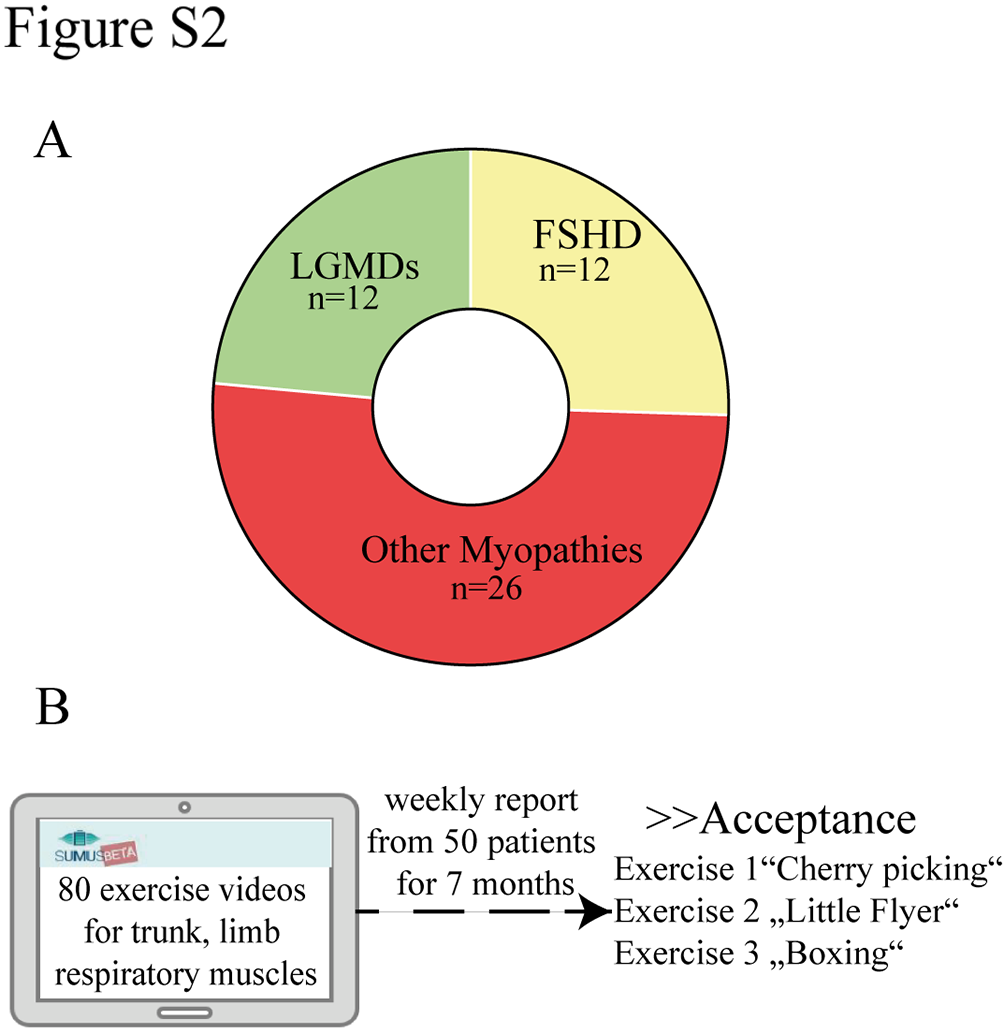

Supplement: Supplementary file 4 [file Image2.TIF]

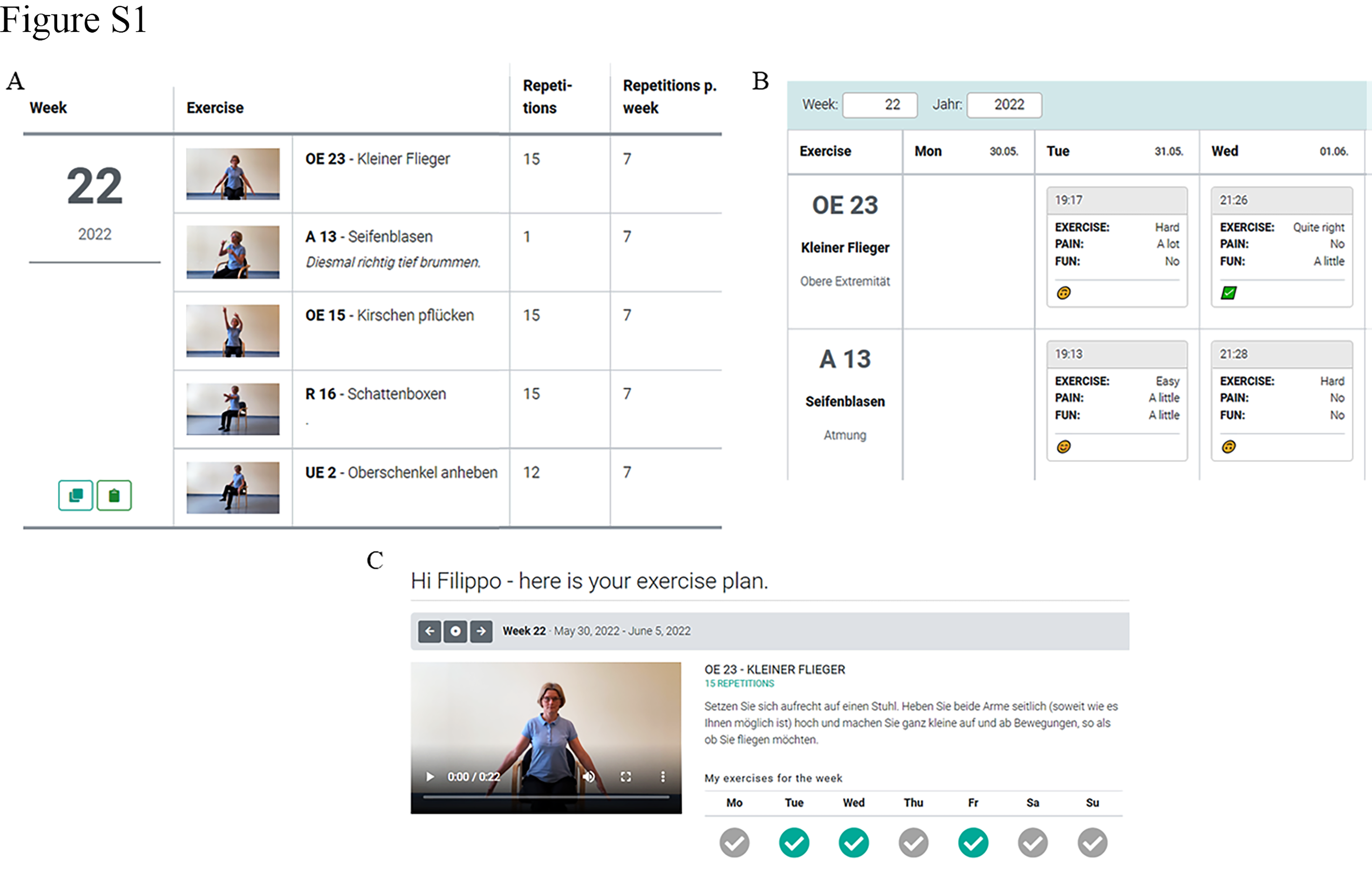

Supplement: Supplementary file 5 [file Image1.TIF]
